# Experiences of Loss, Grief and Support Needs of Adults on Haemodialysis and Families: A Qualitative Explorative Study

**DOI:** 10.1111/jorc.70030

**Published:** 2025-10-04

**Authors:** Jette Marcussen, Rikke Madsen, Ann Bonner, Jette Rude Nielsen, Hanne Agerskov

**Affiliations:** ^1^ Research Unit OPEN, Department of Clinical Research University of Southern Denmark; ^2^ Centre for Nursing, Health Illness and Culture Absalon University College Denmark; ^3^ Faculty of Health Sciences Health Science Research Center, UC University College Lillebaelt Denmark; ^4^ Department of Dermatology Zealand University Hospital Denmark; ^5^ School of Nursing and Midwifery Griffith University Brisbane Australia; ^6^ Kidney Health Service, Metro North Brisbane Australia; ^7^ Department of Public Health Aarhus University Aarhus Denmark; ^8^ Department of Nephrology Odense University Hospital Denmark; ^9^ Department of Clinical Research and Family Focused Health Care Research Centre University of Southern Denmark

**Keywords:** anticipatory grief, family focused support model, haemodialysis, loss

## Abstract

**Background:**

The burdensome life of haemodialysis impacts both patients and their families. It often leads to a stressful life marked by experiences of loss and grief. Nurses report a lack of knowledge and skills in providing grief support, as well as insufficient time for existential conversations with patients and families.

**Objectives:**

To explore the experiences of loss, grief and support needs of both adults on haemodialysis with no option of a kidney transplant and their family members.

**Design:**

A qualitative explorative study, using a phenomenological‐hermeneutical approach and semi‐structured interviews. Ricoeur's interpretation theory was used to analyze data involving three steps: Naïve reading, structural analysis and critical interpretation and discussion.

**Participants:**

Nine adults on haemodialysis and eight family members.

**Findings:**

Haemodialysis treatment alters everyday life for families resulting in experiences of anticipatory grief. Both adults on haemodialysis and their family members experienced challenges with identities and changes in social relationships over the course of the illness with psychological consequences for the family. A holistic family‐focused grief support was identified as an important need for the participants and ought to include time and room for private existential conversations with nurses.

**Conclusion:**

This study offers unique insights into the impact of loss and anticipatory grief in everyday life for those on haemodialysis and their families. Greater existential support and holistic family‐focused grief support is proposed to guide family nursing in kidney care. Healthcare policies and health education in grief ought to reflect the care needs of both patients and family members.

**Baggrund:**

En tilværelse med hæmodialyse påvirker både patienter og deres familier og fører ofte til en stressende hverdag med oplevelser af tab og sorg. Utilstrækkelig psykosocial støtte og manglende familieinvolvering øger yderligere risikoen for patientindlæggelse. Sygeplejersker oplever ofte manglende viden og færdigheder til at yde sorgstøtte, samt utilstrækkelig tid til eksistentielle samtaler med patienter og familier.

**Formål:**

At undersøge oplevelser af tab, sorg og behov for støtte hos voksne i hæmodialyse uden mulighed for nyretransplantation og deres familier.

**Design:**

En kvalitativ, eksplorativ undersøgelse med en fænomenologisk‐hermeneutisk tilgang og semistrukturerede interviews. Ricoeurs fortolkningsteori med tre trin blev anvendt til analyse af data: naiv læsning, strukturel analyse og kritisk fortolkning og diskussion.

**Deltagere:**

9 voksne i hæmodialyse og 8 pårørende.

**Resultater:**

Hæmodialysebehandling forandrer familiernes dagligdag og resulterer i oplevelser af ventesorg. Både patienter og familier oplever udfordringer med identitet og ændringer i sociale relationer gennem sygdomsforløbet. Dette medfører psykologiske konsekvenser i familien. Der er et stort behov for en helhedsorienteret familiecentreret sorgstøtte, der inkluderer tid og rum til private eksistentielle samtaler med sygeplejersker.

**Konklusion:**

Studiet giver unik indsigt i tab og ventesorgs betydning i hverdagen for voksne i hæmodialyse og deres familier. Det anbefales at styrke en familiefokuseret sygepleje til patienter i hæmodialyse og deres familier, samt fremme den eksistentielle støtte og helhedsorienteret familiefokuseret sorgstøtte. Sundhedspolitik og uddannelse i sorgstøtte bør afspejle både patienters og familiens behov.

## 1 Introduction

Globally, approximately 700 million people have chronic kidney disease (CKD), and an estimated 1.2–1.3 million people die from it each year (Bello et al. 2022; Levey et al. 2020; Sundström et al. 2022). When kidney failure (previously termed end‐stage kidney disease) is reached, kidney replacement therapies are needed to sustain life, with most receiving haemodialysis (HD; American Kidney Fund 2023; Bello et al. 2022). Haemodialysis is a burdensome treatment with a significant impact on a person's life such as lifestyle, social life, adherence to dietary and fluid restrictions and to medications, and time spent on HD (Antoun et al. 2022; Caton et al. 2023). Hence, kidney failure and HD can create a stressful situation as well as a loss of a normal life for both the individual and the family (Antoun et al. 2022; Caton et al. 2023; Cha and Han 2020). Some patients and families live with no option of kidney transplantation resulting in fear of death and anger (Frandsen et al. 2020; Moran 2022).

### 1.1 Literature Review

The influence of kidney failure and HD on adults with kidney failure and families' lives often result in experiences of loss including experiences of a “lost life”, grief and unmet needs of support (Frontini et al. 2021; Jones et al. 2018; Monaro et al. 2014). Patients on HD and their family members experience several losses. Often mental and emotional distress are caused by physical loss related to treatment, symptom management, lifestyle restrictions, co‐morbidity, and no option for kidney transplantation (Frandsen et al. 2020; Nataatmadja et al. 2021). The impact of kidney failure and HD on patients and their families also encompasses social losses. The illness, treatment, and symptoms such as fatigue have an impact on the social lives with less time spent with the family (Antoun et al. 2022; Monaro et al. 2014; Yapa et al. 2024). The demands of HD force patients and families to decline social invitations and activities leading to feelings of loneliness and emotional isolation (Nataatmadja et al. 2021). Similarly, informal caregivers and families experience social isolation, and putting their own social lives on hold, which influence their well‐being (Hoang et al. 2018).

The impact of losses and disruptions in everyday life, while on long‐term HD can lead to experiences of grief for both patients themselves (Moran 2022) as well as their families (Barello et al. 2023; Frontini et al. 2021). Emotional overload resulting in grief experiences (Monaro et al. 2014) can include experiences of anticipatory grief during the illness trajectory (Speed 2022). In addition, overwhelming emotions can be a major cause of mental health problems—such as depression and anxiety (Nataatmadja et al. 2021).

The confrontation of death can cause existential issues with anticipatory grief or complications of grief (Harvey 2000; Moran 2022; Speed 2022). Patients on HD and their families have existential difficulties in perceiving a positive future (Monaro et al. 2014; Yapa et al. 2024). Nevertheless, some patients report having a good quality of life, where they can continue to work, be involved in family life, and maintain other significant activities (Antoun et al. 2022; Cha and Han 2020).

Patients and their families report a lack of family support in managing life with HD. A review identified that poor psychosocial support to patients, including lack of family involvement resulted in risk for hospitalization to patients with CKD (Bello et al. 2022). Other studies identified that both patients and families have support needs such as a need for psychological support, emotional validation and improved family coping skills and involvement (Frontini et al. 2021; Sousa et al. 2023).

Previous research of kidney care nurses has identified that loss and grief seem to be present in the lifeworld of patients undergoing HD and their families. However, nurses report having a lack of knowledge and skills to provide grief support to patients, and they also have insufficient time for existential conversations with patients and families (Marcussen et al. 2023; Sedin et al. 2023; Speed 2022). Other studies highlight that patients receiving HD, and their families have been found to have unmet needs for family support from nurses (Frontini et al. 2021; Sousa et al. 2023). The needs become apparent as both patients and families grapple with the consequences of various individual and family losses and grief which could lead to mental health problems (Monaro et al. 2014; Nataatmadja et al. 2021).

Due to the limited knowledge about patients and their families' experiences of the impact of HD, this study sought to explore the experiences of loss, grief and support needs of both adults on HD with no option of kidney transplant and their family members.

## 2 Materials and Methods

This study used a qualitative explorative design (Thirsk and Clark 2017) suitable to explore the perspectives of adults receiving HD and their family members' experiences. A phenomenological‐hermeneutical approach inspired by Ricoeur's theory of narrative and interpretation (Ricoeur 1976) was applied. This approach made it possible to gain in‐depth understanding of the phenomenon through a critical interpretation lens (Ricoeur 1976; Simonÿ et al. 2018). COREQ standards for reporting qualitative research were used (Tong et al. 2007) see Supporting Information File 1.

### 2.1 Participants

Participants were recruited from two HD units at a university hospital in Denmark by a research nurse working in the departments, supervised by a senior researcher responsible for the sampling. Based on the inclusion criteria, the research nurse approached the participants and provided oral and written information about the study. The inclusion criteria were adults receiving HD with no option of having a kidney transplant, and family members of the patient's choice, who were > 18 years of age. Exclusion criteria were cognitive impairment and inability to communicate in Danish or English.

In total, nine patients and eight family members were included in the study (see Table 1). Their experiences from the course of illness varied from four to 40 years. There were five male and four female patients. Two patients lived as singles, five were married and two had a fiancé. Patient ages ranged from 37 to 76 years. Most of the patients did not work due to their kidney failure, or they had retired. There were six female and two male family members. Two of the family members were adult children (females). Five family members were the married partners of the patients and one a fiancé. The age range for family members varied between 19 and 76 years.

**Table 1:** Participants.

Age (years)	Participant characteristics
19–24	2 adult children (family members)
25–34	0
35–44	3 patients
45–54	2 partners (family members)
55–64	2 patients
65–74	3 patients, 2 partners (family members)
75–80	1 patient, 2 partners (family members)

### 2.2 Data Collection

Semi structured interviews informed the study, and a semi‐structured interview‐guide was collaboratively developed by the research team inspired by the literature review and a former study about patients and families' experiences in a nursing perspective. The draft guide was reviewed by a user panel, comprising a patient and a family member from the HD department, resulting in few corrections (Kvale and Brinkmann 2014). Data collection took place between May 2021 and June 2022, and, due to the COVID‐19 pandemic, interviews were carried out through zoom or telephone, based on participants' preferences. In total 17 interviews were undertaken, 13 interviews by three of the authors, who also were experienced female nurses (J.M., R.M., H.A.). These were supplemented with four more interviews to reach the purpose of conducting data sufficiency. The last four interviews were undertaken by a female research assistant in training and supervised by first author (JM). The main interview questions asked were: *Please try to describe what kind of losses you have experienced caused by disease and HD?” And “How does this impact your daily living and experiences (e.g., your emotions and behaviors)? In addition: ‘Please reflect on how those experiences influence your need of support’*. Interview duration ranged from 25 to 60 min, were audio‐recorded and transcribed verbatim (Kvale and Brinkmann 2014).

### 2.3 Data Analysis

**Figure 1:**
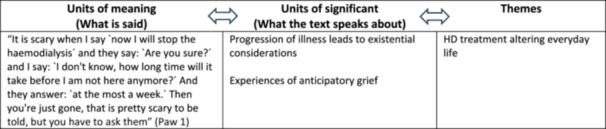
Structural analysis example.

The data analysis was inspired by Ricoeur's interpretation theory on three levels—naïve reading, structural analysis, and critical interpretation and discussion (Ricoeur 1976). The naïve reading consisted of reading the text material several times by all authors to get an initial understanding of what the text was about. The structural analysis was carried out by identifying quotations in the text (‘what is said’) that illuminated the meaning. By questioning the units of meaning (‘what the text speaks about’), a further interpretation was made, which led to the development of themes. Interviews were transcribed and imported into NVivo to support the coding of the data in the structural analysis (QRS International 2019). An example of structural analysis is shown in Figure 1.

The critical interpretation and discussion phase involved drawing from existing studies to obtain a comprehensive understanding of the patients' and families' experiences. The interpretation process involved a dialectic movement between explanation and comprehension going back and forth in the data material (Ricoeur 1976; Simonÿ et al. 2018). The analysis and findings were discussed by the whole research team, which included two senior researchers with extensive clinical experience in kidney care and a third researcher with expertise in loss and grief.

### 2.4 Ethical Considerations

“The Danish Data Protection/Agency SDU RIO” approved the study (number 11.345). The study was also reported to the Regional Committees on Health Research Ethics (number 20212000‐48). Ethical guidelines were followed in accordance with the Helsinki Declaration (WMA 2019) and the Danish Code of Conduct of Research (Ministry of Education and Research. 2015). All participants were informed about the study orally and in writing, and they provided written informed consent. Participants were unknown to the interviewer. Withdrawal was possible at any time, and participants were informed that data would be deidentified to maintain their anonymity.

### 2.5 Findings

The naïve reading revealed that loss and grief impacted the daily living of both patients and families. During HD treatment, patients and families seemed to be challenged by changes in their life resulting in experience of anticipatory grief and existential considerations. There were personal changes in identity and experiences of losing time. Further, family involvement in care was important. Four themes were identified: HD treatment altering everyday life, Challenges with changed identities, Changes in social relationships over the course of illness, and family‐focused grief support was needed. Table 2 presents the themes and subthemes.

**Table 2:** Themes and subthemes.

Themes	Subthemes
Theme 1: HD treatment altering everyday life	Illness has impact in patients and families daily living Experiences of anticipatory grief Progression of illness leads to existential considerations
Theme 2: Challenges with changed identity	Personal changes for all in the family Changes to work identity A changed intimacy between patient and partner
Theme 3: Changes in social relationships over the course of illness	Losing time with important relationships Loss of surrogate family Psychological consequences in families
Theme 4: Family‐focused grief support is needed	A family approach is needed More time and communication are needed Room for private conversation A combination of personal and professional skills

The themes are unfolded as follows. (Pam) refers to patient/man; (Paw) refers to patient/women; (Repw) refers to related partner woman; (Repm) refers to related partner man; and (Rec) refers to related adult child.

### 2.6 HD Treatment Altering Everyday Life

Patients' and families'experiences were often related to restrictions arising from kidney failure and HD treatment. Restrictions influenced their daily living, and the ensuing existential matters related to the seriousness of the disease and its symptoms:

“My greatest loss is my low energy, and that is what I miss most of all; just being normal and living a normal life. My everyday life changes, I have days with loss of function and initiative after HD and I can do nothing”.

(Paw 2)

To family members, life with the illness provided thoughts about anticipatory grief and death of their loved ones. However, the patients and families still wanted to have more time together:

“Well, anticipatory grief… it is lasting…. enormously heavy… the losses you have…. I think it would be easier to cope with, if he was not here anymore, and at the same time I cannot do without him” (Repw 1). Loss was also described as: “when he is home, he is just very tired, you cannot yet completely take time off together” (Rec 1). Another family member expressed: “every second day you crossover in the calendar—she leaves in the morning (for HD), gets home after lunch, and then she is so tired” (Repm 1).

Both patients and family members struggled with the progress of the illness and thoughts about death. The dependency on HD confronted them with existential thoughts related to end‐of‐life issues, indicating a grief process including anticipatory grief. The daily life with critical illness made some patients feel overloaded, coursing existential consequences, deciding to live as a single: “I am living alone, and I do not have children” (Paw 2).

Family members struggled with existential thoughts about their sick family member's future. There were feelings of being ashamed by thoughts about death and at the same time fear of losing a family member:

“I did move out on my own early in my life, because it was just too much, too many memories, seeing your parent just sobbing, that is scary too. There is no closure you are threatened the whole time”.

(Rec 2)

However, patients and family members also focused on the good things in life and the good days they could spend together.

### 2.7 Challenges With Changed Identity

Patients and families were longing to get their previous (normal) life back. They described personal changes, and that a loss of former identity was associated with a loss of possibilities:

“My largest wish is to get my old life back. I thought, why me? I just couldn't understand that I had to be in HD for the rest of my life. You cannot plan; ‘these days, we will go for that’. And that is very heavy. I get tired very fast. If I am changing the beds, then I now need a break, and I cope with that very badly”.

(Paw 1)

The life changes influenced the family identity and patients experienced that they could not keep their former identity of being a supportive parent to their children and family as they used to be: “My two (adult) children live in two other countries. It takes a lot of planning to arrange HD. So, I would say that has probably been the greatest loss for me” (Repw 3). Being dependent on HD also resulted in a changed work identity with the experience of accepting HD as a new job. However, acceptance of having lost the opportunities to work could be a grief process:

“I have been working since I was 13 years old, you need to keep some kind of identity, because if you lose that, then you feel sorry for yourself and that is not good to anyone”.

(Pam 3)

Losing opportunities in life influenced the family and some partners worked part time to cope with an overload because of the family situation. Nevertheless, some turned the situation into a plus by finding meaning in new possibilities, for example, “I have become pretty good to use the Internet, so it is actually me that the others are calling, so that has kind of strengthened my self‐worth” (Pam 4). Both patients and family members invested energy in adapting to the changes and finding meaning in life. The changes also impacted patients' identity as a sexual attractive person: “I did not have a lot of women before, but it is really a kind of loss” (Pam 2). To others, it was related to physical dysfunction:

“I am not able to get erection anymore, so we're not able to have like an old fashion intercourse together. But I like to talk personally. I have talked with one of the nurses about it, but it is a kind of a taboo”.

(Pam 4)

There was a lack of openness in dealing with this topic with partners and health professionals, and to some it led to feelings of loneliness. Sharing experiences about living with the illness within the family appeared to enhance the ability to cope. Conversely, when patients and families were overwhelmed, it was a challenge to share their experience of the loss of normalcy and changed identities.

### 2.8 Changes in Social Relationships Over the Course of Illness

Over the years, the disease and treatment had been causing a loss of social life for both patients and their families; it also meant spending less time with relationships outside the family:

“Being broken in a week and running bad HD, because my body cannot catch up. We must talk about it each time, when we get invited. Do I want to go? Is it worth it? There are things worth it, like visiting my family”.

(Paw 4)

Wider social connections were changed. Most family members did not feel they were part of the life that the patient spent in the HD unit. The treatment was therefore invisible to the family but still had consequences for loss of social relationships in the families. To patients, illness and health problems did consume much of their energy and time resulting in feelings of social loss. Some patients found themselves to be of low value to other people such as being unable to find a new girlfriend: “It is probably more difficult to contact the opposite gender, like having a fiancé again. You don't feel like a good investment. You do not feel like ‘fresh meat on the market´” (Pam 2). Being treated on HD for years provided experiences of having a surrogate family in the ward. There were feelings of loss of close relationships, when co‐patients died, or nurses left for another job:

“I know the nurses and they know me. They are incredibly empathetic, and they see me as a human and not just as a number. It is like having a whole bunch of mothers, and then it is difficult when they leave for new job”.

(Paw 2)

For family members social relationships changed both within and outside the family. Having mental health problems such as anxiety, depression and overload changed the interaction in the family:

“I am scared the whole time (for the death of a parent), and we (the family) have talked it over. Sometimes I feel it's like having PTSD (posttraumatic stress syndrome). My mother hides her feelings, and when my father (on HD) is doing good, then she goes down, she gets depressed, we are all tired, it's a luck that our family did not split up”.

(Rec 2)

Families had experienced critical incidents and engaged in discussions with health professionals about end‐of‐life matters, which led them to worry about each other within the family. The changes in family life caused by illness, its treatment and the consequences of experiencing loss and grief, were often not acknowledged by others outside the immediate families:

“In general, there is a large focus when someone loose a family member to death. Then you are in grief. However, this life with partner on HD is a long‐lasting loss, which is loss of normality”.

(Repw 1)

The changes in family social relationships along with lack of resources to being social outside the family led to an ongoing sense of loss and anticipatory grief. This was often not understood in relationships outside the close family. The lack of capability to manage these challenges provided feelings of overload and mental vulnerability to both patients and families.

### 2.9 Family Focused Grief Support Is Needed

Family members and patients expressed a need for care to be more family inclusive. They believed that nurses should have an active role in supporting the overall well‐being of the entire family to cope with the experience of loss and anticipatory grief:

“The nurses should take a talk with the family concerning what to do. When the HD is not stable, then my family are even more worried at home”.

(Pam 1)

To the families, support and interaction with health professionals were mostly related to the person on HD. Support for the family was important because patients and families acted protectively towards each other:

“Children protect us as parents; they don't tell us everything. We should also have groups only for children or for young people, where they could talk. Children do not have the same on their heart, as a grown‐up has”.

(Repw 2)

The quotation above illustrates how parents felt that younger family members could benefit from being supported by group sessions. In general, family members wished there was time for conversations with a focus on both individual family members and the family together. For family members, it was difficult to find professional support outside of the HD department:

“We did not fit in anywhere, it is not normal being a patient in HD, being at the same time seriously ill and reasonably well functioning”.

(Repw 1)

Having a personal relationship with a nurse was significant when patients felt a need to share experiences related to loss and grief. Patients recognized that their family needed support to cope with loss and grief:

“It is what is needed, to get it processed, and you can see there is not much time for talking, but it is a big part of coping with your illness, that we talk, at least with the nurses. So, we as patients need to talk and the family also needs to talk”.

(Pam 3)

To family members, conversations with nurses were few and mostly related to the patient's health condition. Nevertheless, patients and family members experienced a need for a private room for conversations about loss and grief:

“Could you have a room where you could come or where families could stay or maybe just walk in and sit down and have a cup of coffee, or where there could be a nurse coming and talking”.

(Repw 1)

There was a need for support that could cover experiences of grief. However, it was considered important that support included both personal and professional qualities:

“A basic warmth and care are needed, and when you express vulnerability or are down, then a sense of getting taken care of, with a loving hand and sometimes also humour. There is a need of a very solid human factor”.

(Pam 2)

Family‐focused grief support was considered important to both patients and families. Feelings of being supported during experiences of loss and grief in the illness trajectories was addressed in terms of nursing skills with emphasis on for example, family care, talks, empathy, and humor.

## 3 Discussion

The present study brings to light the emotional overload experienced by patients on HD with no option of a kidney transplant and their families, revealing profound challenges tied to loss of daily life, identity shifts, and disruptions in social relations. The study also showed an unmet need for holistic family nursing grief support to cope with the experiences of loss and grief, and to promote well‐being in families.

This study found several challenges faced by patients living with kidney failure when receiving HD and their families. Both experienced a burdensome life with loss of energy, changed identities, social consequences, and mental health problems such as anxiety, depression, and emotional overload resulting from experiences of loss and anticipatory grief. These challenges have also been identified in other studies (Monaro et al. 2014; Nataatmadja et al. 2021; Yapa et al. 2024). Several studies emphasize that the mental health for patients on HD is frequently compromised, manifesting in feelings of anger, depression, and hopelessness (Antoun et al. 2022; Moran 2022; Rebollo Rubio et al. 2017). Partners of patients on HD also grappled with pervasive emotions such as sadness, resentment, guilt, and a profound sense of loss (Monaro et al. 2014; Speed 2022). Like other studies, findings from the present study showed that the disease not only impacted individuals physically, but also significantly influenced their mental well‐being, existential concerns, and relationships to close family members (Bello et al. 2022; Nataatmadja et al. 2021; Sousa et al. 2024). Such findings are also highlighted as a multifaceted nature of the challenges that patients and their partners encounter throughout life with kidney failure (Sousa et al. 2021). These perspectives emphasize a need for a nuanced and comprehensive approach in family support throughout the HD journey with multidisciplinary renal teams including social workers or social nurses and psychologists.

The study showed experiences of anticipatory grief and stress overload in family members. These findings could indicate symptoms of the grief diagnosis termed prolonged grief disorder by the WHO (World Health Organization 2018). Prolonged grief is characterized by an intense and persistent sorrow and can further complicate mental health challenges as family members struggle to cope with ongoing stress or caregiver burden (Lu et al. 2024; Sousa et al. 2024; World Health Organization 2018). Though, the diagnosis of prolonged grief disorder is made to identify and treat bereaved persons after having lost a loved one by death, the symptoms of prolonged grief disorder have been identified in studies with caregivers expecting death or living with a heavy caregiver burden (Nielsen et al. 2017; Sardella et al. 2023). This study, however, also identified that spending time with an ill family member gave meaning to the family members even though the end of life may or may not have been imminent. A systematic review identified that caregivers could experience personal growth and develop resilience to continue caring for their loved one (Hoang et al. 2018). Another study showed that the experience of purpose in life was associated with positive flexible coping strategies and acceptance coping of the caregiver burden (Sousa et al. 2024).

Studies have emphasized that it is important that nurses have knowledge and skills to prevent prolonged grief disorder (Mauro et al. 2018; Nielsen et al. 2017; Speed 2022). Acknowledging the impact of CKD and HD on families is crucial for creating interventions that reduce caregiver burden and enhance patient and family support. The present study showed that family members with a family member without hope for kidney transplantation experienced a heavy caregiver burden. Other studies have found that families who support family members on HD, face considerable caregiver burden and a tendency to neglect their own health (Hoang et al. 2019; Nataatmadja et al. 2021; Nielsen et al. 2017; Sousa et al. 2024). This underscores the need for comprehensive support structures not only for patients, but also for families (Frandsen et al. 2023; Sedin et al. 2023).

**Figure 2:**
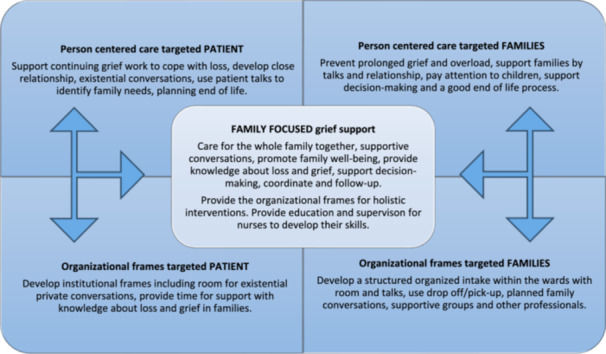
A model of holistic family‐focused grief support. The arrows symbolize the importance of incorporating knowledge from both the patient's perspective and the family's perspective when bringing the entire family together.

Nurses in HD wards work closely with patients and are responsible for providing care and meeting the patients' overall needs. However, providing family focused grief care support requires implementation of strategies with focus on the entire family and that align with nurses' skills (Barello et al. 2023; Frontini et al. 2021; Marcussen et al. 2023; Sousa et al. 2023) Findings from the current study highlight the importance of implementing a holistic family grief approach. This approach is presented as The Model of Holistic Family Focused Grief Support (see Figure 2). The model aims to support health professionals in clinical practice to develop comprehensive care by viewing the whole family at its core and applying person‐centered care to target patients and family members. The approach emphasizes the importance of promoting ongoing grief work and preventing mental health complications including prolonged grief disorder, and providing guidance to cope with loss, anticipatory grief, and existential issues.

Key components of such an approach are also found in other studies emphasizing the importance of grief supportive conversations, involving the entire family and equipping them with needed knowledge to cope with their loss and grief processes (Kissane et al. 2006; Kokorelias et al. 2019). Thus, nurses have a crucial role in facilitating family grief support and in coordinating and follow‐up during the illness trajectory, bringing the family together. In many countries, renal teams are multidisciplinary and often have social workers and psychologists as team‐members who support patients and family members (Cheung et al. 2021). This, however, is not the situation in Denmark where it is the responsibility of nurses to provide the psychosocial healthcare for patients and families' needs. Furthermore, we were unable to identify holistic family focused grief care interventions in the context of CKD and/or HD, although other studies in different health contexts exist (Kokorelias et al. 2019; Madsen et al. 2022; Rancour and Brauer 2003).

This study has notable strengths and limitations. It was guided by Lincoln and Guba's criteria of credibility, transferability, dependability, and confirmability (Lincoln and Guba 1985) which serve to enhance the study's overall trustworthiness: The research team took collective responsibility for maintaining quality and transferability and dependability throughout the study's design, data collection, and data analysis. Following Morse's recommendations, the team ensured trustworthiness through a systematic approach in applying methods and interpreting findings, considering transparency at every stage (Morse 2015). Another strength is the involvement of a senior researcher connected to the wards, but unfamiliar with the participants, contributing valuable insights to the study. Regarding limitations, the inclusion of participants from the same families and only in Danish context may limit the transferability to other countries. However, the study does provide in‐depth insight and confirmability into family perspectives that to some extent could be transferable to other countries and patients/families with similar challenges. Another limitation is that four researchers undertook interviews although the use of an interview guide and the robust analytical coding of data assisted with credibility of the study. Throughout the study, the research team discussed data collection between each interview to purposively recruit and to capture wider perspectives. All participants, however, were ethnic Danes and findings may not reflect the multicultural diversity of the Danish HD population. Regarding the anonymity of the participants, they were not told that they would be quoted directly, and this could be relevant in the future, however they were told that they would be anonymized. Due to the COVID‐19 pandemic, the participants could choose to be interviewed online or via telephone which may have influenced the depth and quality of the data, although conducting interviews through technological solutions allowed the research to continue despite the challenges posed by the pandemic (Richmond et al. 2022; Salanti et al. 2022).

### 3.1 Implications for Clinical Practice

The findings collectively underscore the importance of addressing not only the physical and technical aspects of HD treatment, but also the psychological, social and existential needs of both patients and families in nursing practice. Nursing care should also include continuing grief work and prevention of prolonged grief disorders in family members. Providing family‐focused grief support, which includes existential conversations, requires establishment of close relationships between nurses and families (Marcussen et al. 2023). The proposed model may assist all members of the kidney care team to take this holistic view. Lastly, healthcare policies and health education in grief ought to be developed and reflect the support needs of patients and family members.

## 4 Conclusion

Both patients on HD with no option for a kidney transplant and families experience considerable loss and grief as disease and treatment substantially alters everyday life. There is a loss of identity and social life for both patients and family members. Loss and grief are likely to add to mental health and well‐being challenges. As such interventions for the whole family are needed and we propose an approach of holistic family‐focused grief model support to inform kidney care. Such an approach addresses not only the physical aspects of the patient's disease and treatment but could also assist with supporting the existential challenges with anticipatory grief. Further research to develop and evaluate a family loss and grief intervention informed by the family grief model is required. Interventions could be targeted to reduce symptoms of anticipatory grief, prolonged grief and mental health problems, and thus improve the well‐being of both patients on HD and their families.

## Author Contributions

**Jette Marcussen:** principal project leader, project administration, funding, design of study, data collection, data analysis, software/NVivo, writing original draft, revision and final editing and approval of the manuscript. **Rikke Madsen:** design of study, data collection, data analysis, writing, critical revision and final approval of the manuscript. **Ann Bonner:** design, data analysis, critical revision and final approval of the manuscript. **Jette Rude Nielsen:** design, data collection, writing and final approval of the manuscript. **Hanne Agerskov:** design of the study, data collection, data analysis, critical revision and final approval of the manuscript, supervision.

## Ethics Statement

Participation in the study was voluntary and confidentiality was assured. Ethical approval was obtained from Regional Committees on Health Research Ethics, Denmark.

## Conflicts of Interest

The authors declare no conflicts of interest.

## Supporting information

sup file 1 COREQ_20.8.25.

## Data Availability

A coding book can be provided on request.
